# Androgen receptor modulatory miR-1271-5p can promote hormone sensitive prostate cancer cell growth

**DOI:** 10.3389/fonc.2024.1440612

**Published:** 2024-08-29

**Authors:** Foteini Kalofonou, Damien A. Leach, Sue M. Powell, Jonathan Waxman, Claire E. Fletcher, Charlotte L. Bevan

**Affiliations:** Androgen Signalling and Prostate Cancer Laboratory, Imperial Centre of Translational and Experimental Medicine, Department of Surgery and Cancer, Imperial College, London, United Kingdom

**Keywords:** microRNAs (miRs), antisense oligonucleotides (ASOs), apoptosis, enzalutamide, prostate cancer

## Abstract

In most patients with advanced prostate cancer treated with hormonal therapy, androgen independence eventually emerges, leading to death. Androgen receptor signalling remains an important prostate cancer driver, even in the advanced disease stage. MicroRNAs (miRs), non-coding RNAs that regulate gene expression by inhibiting translation and/or promoting degradation of target mRNAs, can act as tumour suppressors or “oncomiRs” and modulate tumour growth. Because of their stability in tissues and in circulation, and their specificity, microRNAs have emerged as potential biomarkers, as well as therapeutic targets in cancer. We identified miR-1271–5p as an androgen receptor modulatory microRNA and we show it can promote hormone sensitive prostate cancer cell growth. Inhibition or overexpression of miR-1271–5p levels affects prostate cancer cell growth, apoptosis and expression of both androgen receptor target genes and other genes that are likely direct targets, dependent on androgen receptor status, and tumour stage. We conclude that miR-1271–5p has the potential to drive progression of hormone-dependent disease and that the use of specific inhibitors of miR-1271–5p may have therapeutic potential in prostate cancer.

## 1 Introduction

Prostate Cancer (PCa) is the most common type of male cancer and the second leading cause of cancer related death among men in the UK (1–3). PCa is driven by the androgen-signalling pathway; androgens are required for prostate development, growth, function and they mediate signalling through the intracellular protein, the androgen receptor (AR) (4, 5). AR is the major therapeutic target in PCa, but resistance to AR and androgen signalling-targeting drugs eventually occurs and this therapy resistant, advanced stage is usually lethal. Digital Rectal Examination (DRE) and prostate specific antigen (PSA) testing are currently used in the diagnosis of PCa (6, 7), usually together with transperineal or transrectal prostate biopsy (7, 8). Imaging with the use of multi-parametric MRI can have an important role reducing overdiagnosis of clinically insignificant PCa and improving detection of clinically significant cancer (9). PSA is the most widely used biomarker in the PCa diagnostic pathway, however its lack of specificity can lead to overdiagnosis and overtreatment (10). Due to the limitations of current diagnostic markers and treatment strategies, there is a great need for both new biomarkers and therapeutic targets for PCa.

MicroRNAs (MiRs) are small non-coding RNAs that regulate gene expression post-transcriptionally, mainly through inhibition of translation and/or promotion of mRNA degradation, interacting most frequently via the 3’ untranslated region (3’UTR) of mRNA targets (11). MiRs are considered to be master regulators of gene expression, modulating the expression of most protein-coding genes, via interactions between the ‘seed’ sequence of miR and the complementary sequence in the target mRNA, with the majority of mRNAs having many miR binding sites (11, 12). MiRs are secreted by tumours and can be detected in the circulation, and they are widely considered to have major potential as diagnostic, prognostic and predictive biomarkers (13, 14).

Furthermore, certain miRs have been shown to act as tumour suppressors, suppressing tumour growth, or as oncogenes (oncomiRs), promoting it, making them appealing therapeutic targets (13, 15). Levels of miRs can be increased using miR mimics, chemically modified RNA duplexes that enhance levels of endogenous mature miRs, while antisense oligonucleotides (ASOs) can be applied to reduce levels of mature miRs via miR-sequestering, through seed complementarity (16). In this study, we investigated the relevance to PCa of miR-1271–5p, encoded by a gene located in chromosome 5 (5q35.2). The role of miR-1271–5p is complex and differs in various types of cancer (17, 18). In this study, we investigate the effect of miR-1271–5p on the AR pathway, aspects of PCa growth and the potential role of miR-1271–5p in PCa treatment.

## 2 Results

### 2.1 MiR-1271–5p modulates AR activity in PCa cells

**Figure 1:**
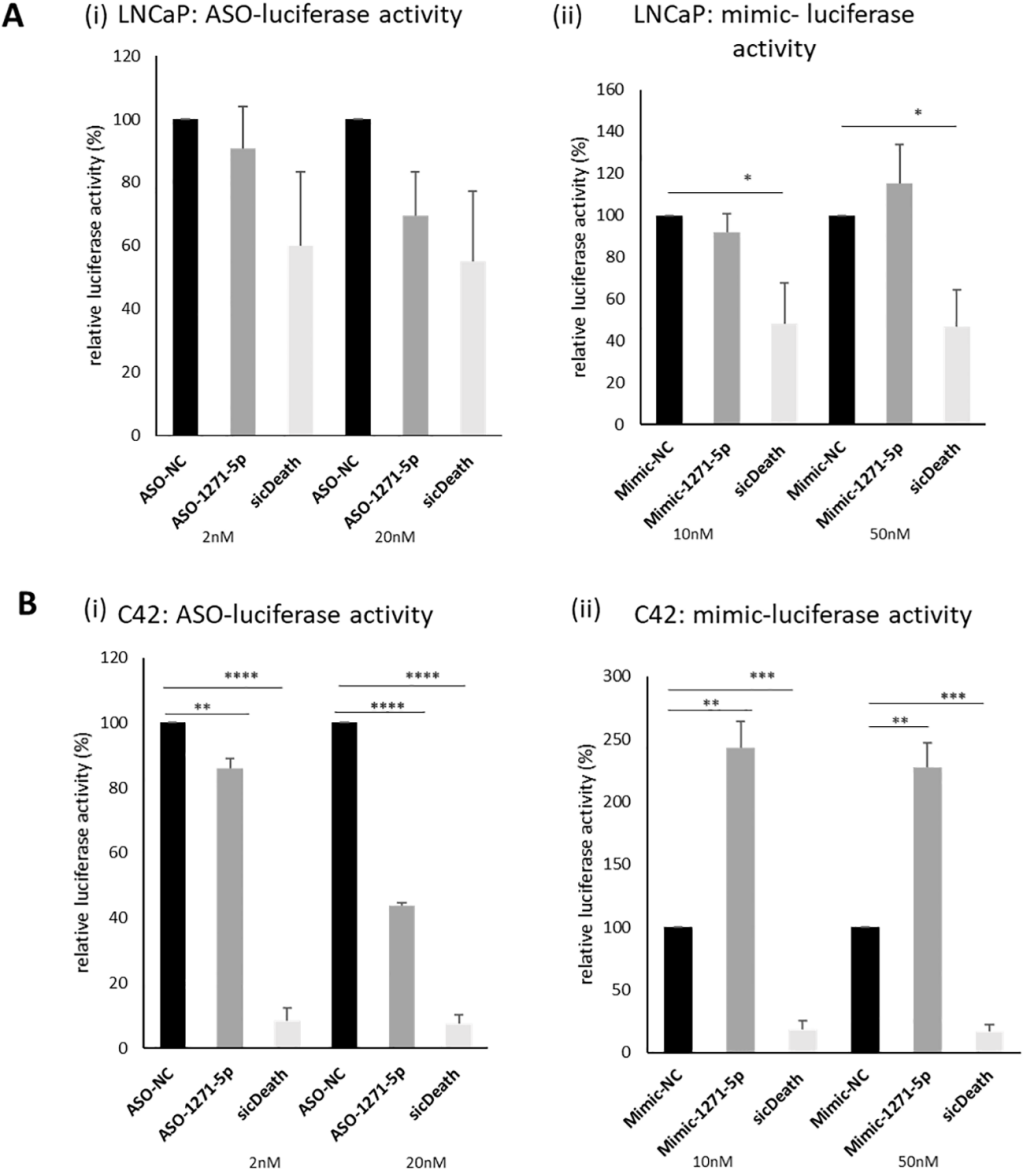
Effect of ASO-1271-5p and Mimic-1271-5p on AR-luciferase activity of LNCaP and C42 cells. Luciferase activity of the effect of miR-1271-5p in LNCaP **(A**i, ii**)** and C42 cell lines **(B**i, ii**)**, containing an AR reporter-luciferase vector (MAR4). Luciferase assay was performed 48h post transfection. A non-targeting miR inhibitor and mimic were used as a negative control (NC) and sicDeath as positive control for normalisation. Data represents mean relative to NC luciferase activity of three independent experiments, performed in duplicate + SEM, corrected for cell number. Statistical analysis was performed by an unpaired (two sample) Student's t-test (two tailed). *P ≤ 0.05, **P ≤ 0.01, ***P ≤ 0.001, ****P≤ 0.0001. Significance is shown compared to NC at the appropriate concentration.

An initial high-throughput miR inhibitor screen was previously conducted, using a library of antisense oligonucleotide miR inhibitors (ASOs), transfected into cell line models of both androgen responsive (LNCaP) and castrate resistant (C42) PCa cell lines, carrying an AR reporter vector (19). MiR-1271–5p was selected for further validation, based on its effects on AR activity in both cell lines. The castrate resistant C42 cell line, which is derived from LNCaP, differs from the parent line in expressing lower levels of AR protein and mRNA transcript (14). In validation experiments, ASO-1271–5p indeed reduced activity of an AR-driven luciferase reporter gene in both lines, non-significantly in the AR positive LNCaP **Figures 1Ai,ii**) and significantly so in the castrate resistant C42 cell line (**Figure 1Bi ii**), at both 2nM (p=0.0079) and 20nM (p<0.0001), with a concentration-dependent increase in effect. Conversely, in the C42 cells, increasing levels of miR-1271–5p by transfecting in a mimic significantly increased AR activity, at both low (10nM, p=0.0024) and high (50nM, p=0.0029) concentrations, 48h post transfection, normalised for cell number. This corroborates previous results (19) and shows that ASO-1271–5p reduces AR activity, which is opposite to the effect of the Mimic-1271–5p. Effect of ASO-1271 and Mimic-1271–5p on mRNA levels of AR and its target genes are found on supplementary data (**Supplementary Figure 1**).

### 2.2 MiR-1271–5p affects growth of PCa cell lines

**Figure 2:**
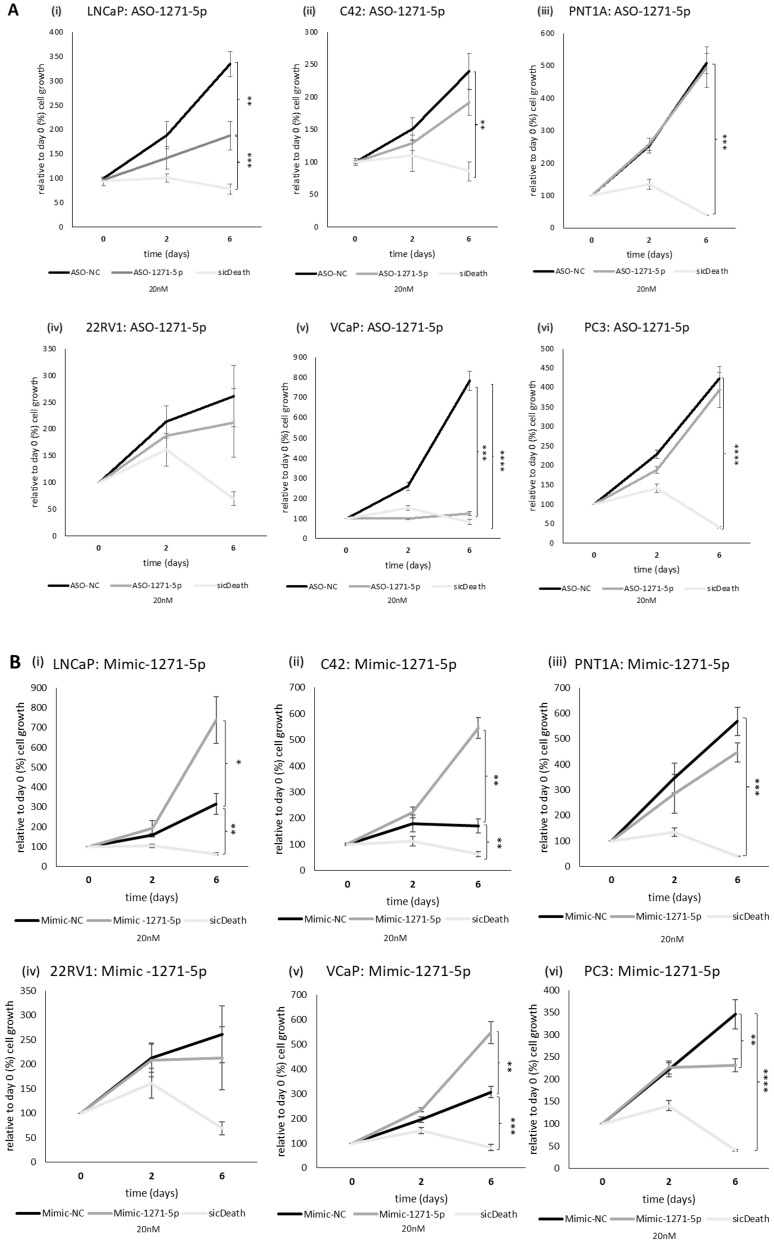
Effect of miR inhibition or overexpression on cell growth of PCa cells using an SRB assay: (i) LNCaP, (ii) C42, (iii) PNT1A, (iv) 22RV1, (v) VCaP and (vi) PC3 cells were transfected with **(A)** ASO-1271-5p or **(B)** Mimic-1271-5p at 20nM concentration and three different time points were selected: day 0, 2, 6. Cell growth was measured 48h post transfection, relative to the percentage (%) of day 0 values. A non-targeting miR inhibitor and sicDeath were used as negative and positive controls, respectively. Cells were grown in stripped RPMI media-5% CSFCS, supplemented with 0.1nM MB. Data represents mean absorbance of three independent experiments performed in quadruplicate + SEM. Statistical analysis was performed by an unpaired (two sample) Student's t-test (two tailed). *P≤0.05, **P ≤ 0.01, ***P≤ 0.001, ****P≤ 0.0001.

Given the effect observed on AR activity, we examined the role of miR-1271–5p on the growth of a range of PCa cell lines, representing different AR status (see **Supplementary Table 1**), using an SRB assay. SicellDeath (sicDeath, Qiagen), a pool of siRNAs that knock-downs various genes essential for cell survival, was used as a positive control for growth inhibition (20). ASO-1271–5p significantly reduced cell growth of androgen sensitive/AR-positive cell lines LNCaP and VCaP, when compared to a non-targeting control (ASO-NC) [LNCaP (p=0.0099) and VCaP (p=0.00019), (**Figures 2A**i, v)]. The opposite, significant effect was seen when LNCaP, C42 and VCaP cell lines were transfected with Mimic-1271–5p [(LNCaP: p=0.0299, C42: p=0.0241, VCaP: p=0.0126), (**Figures 2B**i, ii, v)], with increased proliferation at six days (in keeping with the effect on AR reporter activity in LNCaP and C42 cells with the addition of miR inhibitor or mimic). No significant effect was observed on 22Rv1 cells, with either the use of ASO or mimic (p=n.s., **Figures 2A**iv, **B**iv). Importantly, growth inhibition by ASO-1271–5p was not seen in the AR-negative PC3 cells (p=n.s.), nor in the non-malignant PNT1A cells (p=n.s.), however in the PC3 cell line, there was small but significant reduction of cell growth with the Mimic-1271–5p (p=0.0192), compared to NC (**Figure 2B**vi).

Therefore, it seems that ASO-1271–5p has different effect on cell growth of PCa cell lines based on AR status, in AR-positive lines it has an inhibitory effect and the effects on proliferation reflect the effects on AR activity. In cell lines representing CRPC and metastatic disease (such as PC3 cells), ASO-1271–5p has no, or minimal/non-significant effect on cell growth, while the mimic tends to reduce the cell growth.

### 2.3 Effect of enzalutamide addition to ASO-1271–5p or Mimic-1271–5p on LNCaP cell growth

**Figure 3:**
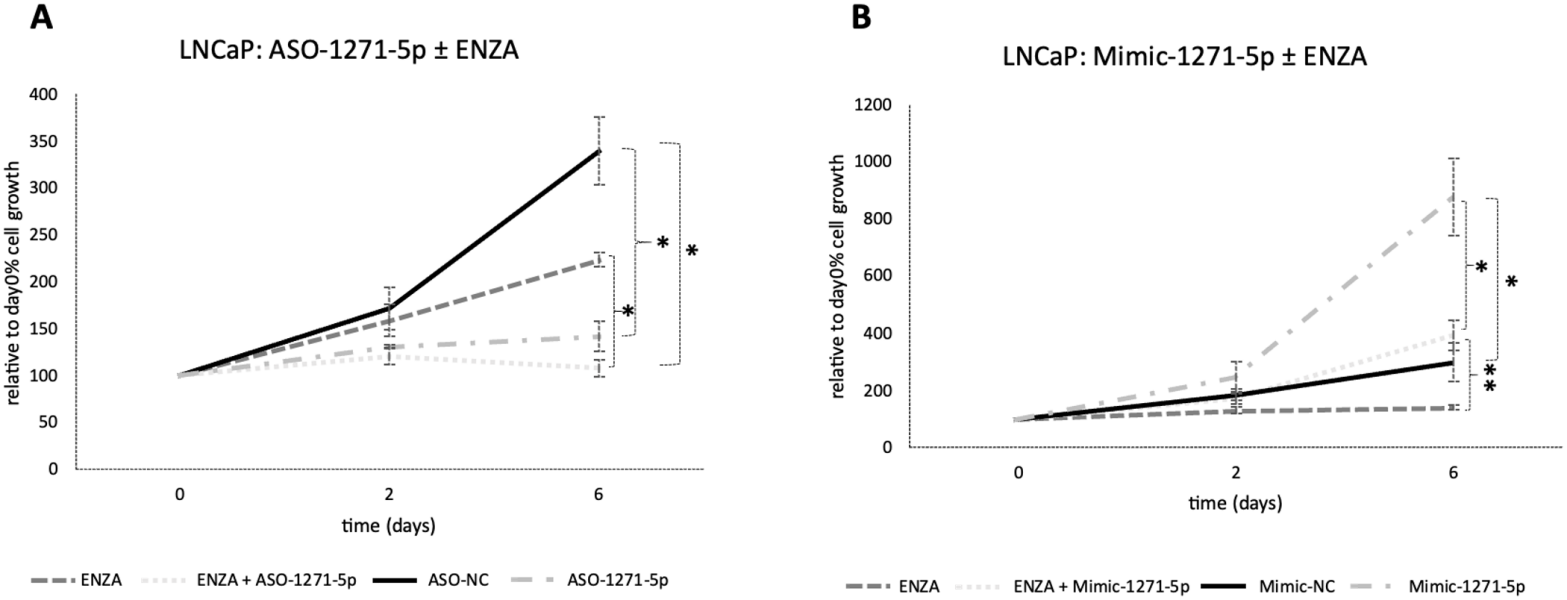
Addition of enzalutamide to ASO-1271-5p dramatically reduced LNCaP cell growth and partially abrogated the effect of the mimic. LNCaP cells were transfected with enzalutamide (ENZA) at 0.8 uM and ASO-1271-5p or Mimic-1271-5p at 20nM. Cell growth was measured 48h post transfection, relative to the percentage (%) of day 0 absorbance and three different time points were examined: day 0, 2, 6, via an SRB growth assay. NC inhibitor or mimic was used, as well as 0.8% v/v DMSO. Data represents mean absorbance of three independent experiments performed in quadruplicate + SEM. Statistical analysis was performed by an unpaired (two sample) Student's t-test (two tailed). *P≤0.05, **P≤0.01.

To determine whether the addition of enzalutamide, a commonly used hormonal therapy, would increase the efficacy of cell growth inhibition in combination with miR targeting, the androgen-responsive and enzalutamide-sensitive LNCaP cells were transfected with ASO-1271–5p or Mimic-1271–5p and they were also treated with enzalutamide. Addition of enzalutamide to cells transfected with ASO-1271–5p produced an additive, significant reduction of cell growth, compared to enzalutamide alone (p=0.021) (**Figure 3A**). Predictably, addition of enzalutamide to Mimic-1271–5p reduced the extent of growth upregulation elicited by Mimic-1271–5p alone (p=0.007) (**Figure 3B**). Overall, this indicates that there is a potential role for combination treatments with ASO and enzalutamide, since the addition of ASO-1271–5p led to a further reduction of LNCaP cell growth compared to enzalutamide alone.

### 2.4 Effect of miR-1271–5p modulation on apoptosis

**Figure 4:**
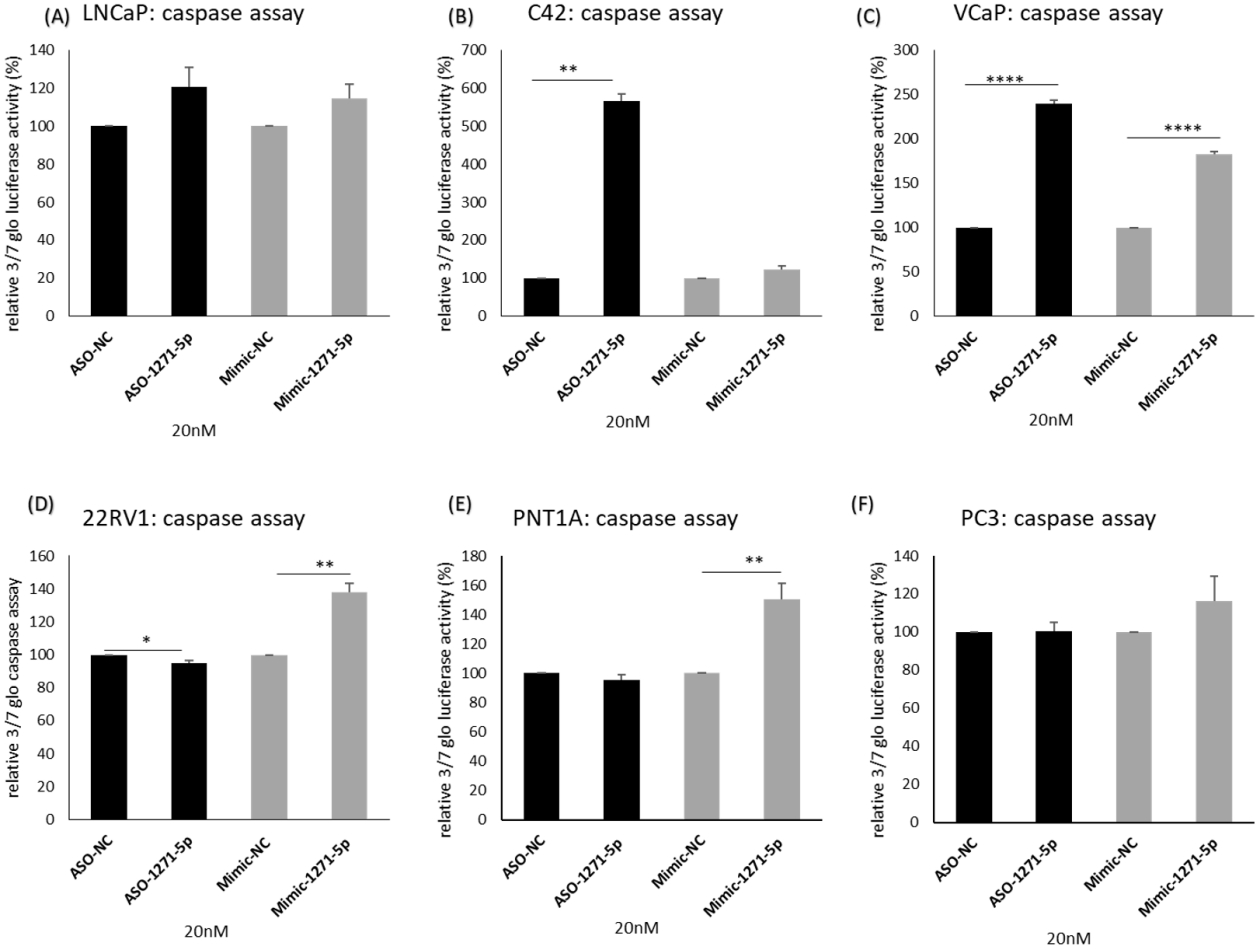
Effect of miR-1271-5p inhibition or overexpression on apoptosis. A caspase 3/7 glo assay was performed to verify the effect of miR-1271-5p inhibition or overexpression on apoptosis of **(A)** LNCaP cells, **(B)** C42 cells, **(C)** VCaP cells, **(D)** 22RV1 cells, **(E)** PNT1A cells and **(F)** PC3 cells. Caspase assay was performed 72h post transfection. Data represents mean absorbance of three independent experiments performed in quadruplicate + SEM. Statistical analysis was performed by an unpaired (two sample) Student's t-test (two tailed). *P≤ 0.05, **P≤0.01, ***P≤ 0.001, ****P<0.0001.

To further elucidate the mechanisms of miR-1271–5p, the effects of inhibition or overexpression on cell death were investigated (**Figure 4**). In both C42 and VCaP cells, ASO-1271–5p significantly and markedly increased apoptosis (p=0.0017 and p<0.0001 respectively) (**Figures 4B, C**), but it marginally decreased apoptosis (p=0.0496) in 22RV1 cells (**Figure 4D**). Interestingly, Mimic-1271–5p also significantly increased apoptosis in VCaP cells (p<0.0001), but to a lesser extent compared to the effect of ASO (**Figure 4C**). Mimic-1271–5p significantly increased apoptosis in 22RV1 (p=0.0028) (**Figure 4D**) and PNT1A cells (p=0.0035) (**Figure 4E**), which could reflect the differences in AR expression levels and miR regulation. Overall, we saw that the addition of ASO-1271–5p is leading to reduction of cell growth and increase of apoptosis, most significantly in VCaP cells, again supporting a potential therapeutic role of miR-1271–5p inhibition.

### 2.5 Effect of miR-1271–5p modulation on mRNA levels of miR target genes

We further investigated the effect of miR-1271–5p on expression levels of candidate target genes. A high-throughput PAR-CLIP analysis (21) identified a panel of genes that are targeted by miR-1271–5p, which include *APPL1, MORF4L1, TMBIM6, SND1, ELK4* and *SPEN*. These were selected for further investigation, as they are protein-coding and had the highest number of total occurrences, representing the highest number of potential binding sites for miR-1271–5p, mainly within the 3’ UTR.

**Figure 5:**
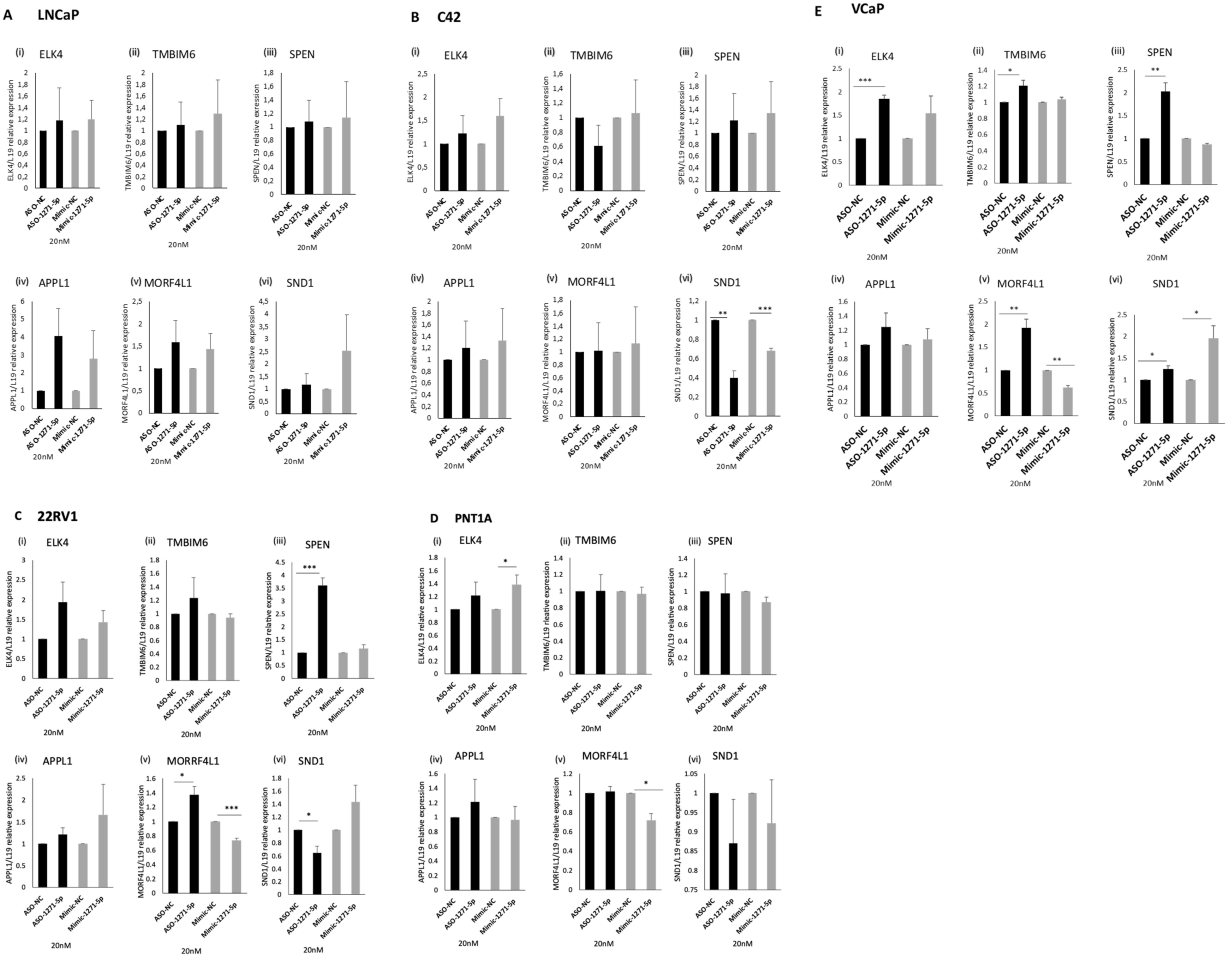
Effect of miR-1271-5p inhibitor and mimic on mRNA levels of miR-1271-5p target genes. RT-qPCR analysis of (i) ELK4, (ii) TMBIM6, (iii) SPEN, (iv) APPL1, (v) MORF4L1, (vi) SND1 in **(A)** LNCaP/MAR4, **(B)** C42/MAR4, **(C)** 22RV1, **(D)** PNTIA **(E)** VCP cells transfected with 20nM of ASO-1271-5p or Mimic-1271-5p. Cells were transfected with miR-ASO/mimic 24h post seeding and they were further incubated for 48h after the transfection. A NC was used for each experiment. L19 was used for normalisation. Data represents mean relative expression of three independent experiments performed in triplicate ± SEM. Statistical analysis was performed by an unpaired (two sample) Student's t-test (two tailed). *P≤0.05, **P≤0.01, ***P≤ 0.001.

To verify the effect of miR modulation on mRNA expression levels of these genes, ASO-1271–5p and Mimic-1271–5p were transfected into a panel of PCa cell lines and RT-q-PCR analysis was conducted (**Figures 5A–E**). For a *bona fide* direct mRNA target of miR-1271–5p, we would expect that the addition of ASO would result in an increase of mRNA levels while transfection with the mimic would decrease mRNA levels. SPEN and MORF4L1 demonstrated this in 22RV1 [SPEN (p=0.000832) and MORF4L1 (p=0.0351)] and VCaP cells [SPEN (p=0.0056), MORF4L1 (p=0.0088)], as transfection of ASO-1271–5p significantly upregulated their mRNA levels (**Figures 5C, E**). The addition of Mimic-1271–5p significantly reduced MORF4L1 mRNA levels in the case of 22RV1 (p=0.001067) (**Figure 5C**), PNT1A (p=0.01564) (**Figure 5D**) and VCaP (p=0.0017) (**Figure 5E**) cells. This suggests that MORF4L1 acts as a conventional downregulated mRNA target. Similarly, ELK4 and TMBIM6 are potential *bona fide* miR-1271–5p target genes in PCa, as in VCaP cells, ASO-1271–5p significantly increased their mRNA levels [ELK4 (p=0.00049), TMBIM6 (p=0.0387) (**Figure 5E**)]. However, SND1 does not appear to be conventionally regulated, since, while miR-1271–5p overexpression significantly reduced SND1 levels in C42 (p=0.0014), so too did ASO-1271 (**Figure 5B**), while conversely increasing SND1 in VCaP cells (**Figure 5E**).

Overall, ASO-1271–5p significantly increased target gene expression in VCaP cells, a cell type which is androgen responsive and expresses wild-type AR, suggesting conventional miR downregulation. Different cell lines respond differently to miR-1271 modulation, which could be at least in part due to their differing endogenous miR levels (**Supplementary Figure 2**), transfection efficiency in cell lines and miR targeting. Interestingly, this mixed response of genes to modulation of endogenous miR-1271–5p levels in different cell lines could reflect the dual role of miRs in upregulation and downregulation of target genes, as well as their distinct role at different stages of PCa progression.

### 2.6 Expression of miR-1271–5p target genes in PCa tumours

SND1 was previously shown to be a validated miR-1271–5p target gene by AGO2-PAR-CLIP analysis (21). This was selected for further investigation, based on antibody availability, staining levels according to the Human Tissue Atlas and publicly available datasets. TCGA data were analysed to verify correlation between SND1 and miR-1271–5p (Pearson correlation = 0.016. P value =0.715), indicating SND1 being a non- conventionally downregulated target, as demonstrated in previous results of miR-1271–5p modulation on alteration of mRNA levels of SND1 (section 2.5).

**Figure 6:**
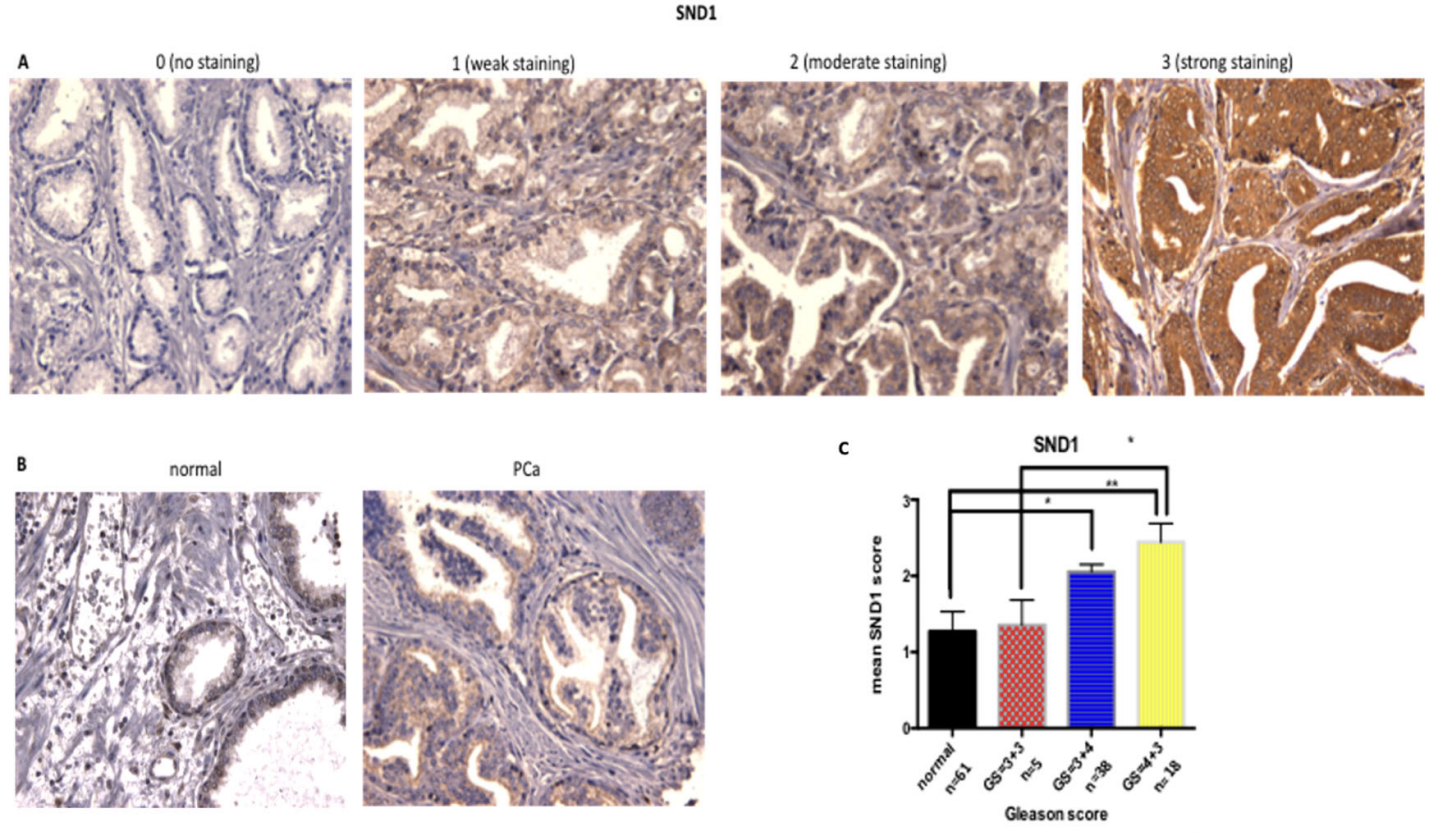
Increase of SND1 staining in patients with higher GS. **(A)** Examples of SND1 immunohistochemistry in epithelial cells using the quick score method as: 0 (negative), 1 (weak), 2 (moderate), 3 (strong) staining. **(B)** Normal and cancer sections from the same patient were stained for SND1. **(C)** Statistical analysis of the mean SND1 staining intensity, based on the 0-3 scoring system, relative to Gleason Score. Normal: n = 61, GS = 3 + 3 : n = 5, GS = 3 + 4 n = 38 , GS = 4 + 3 : n = 18 Three different images were taken per tissue, per patient, scored by two independent assessors. Statistical analysis was conducted using one-way ANOVA. *P ≤ 0.05 **P ≤ 0.01, ***P ≤ 0.001 , ****P≤0.0001.

To determine whether SND1 was altered in PCa tissue of different Gleason Score (GS), protein levels were assessed in a cohort of 61 PCa patients. SND1 expression in tissue was based on intensity of staining, using the quick score method [(0): no staining to (3): strong staining for SND1 antibody (**Figures 6A–C**)].

Overall, SND1 levels were higher in cancers compared to matched normal tissue, and levels increased with grade, with the Gleason grade 7 cases being divided into GS=3 + 4 and the more aggressive GS=4 + 3. SND1 exhibited significantly higher staining in GS=3 + 4 tissue compared to normal (p=0.0137). GS=4 + 3 had the highest SND1 staining, compared to 3 + 3 and 3 + 4 tissues, with the former comparison reaching significance (p=0.0151) (**Figure 6C**).

Examples of weak to moderate staining, according to the 0–3 scoring system in three distinct examples of tissues stained with APPL1 are shown in **Supplementary Figure 3**. In the case of normal tissue stained with the antibody against APPL1, compared to cancerous tissue, derived from the same patient, there was more intense staining in the case of the cancer tissue. Statistical analysis of groups of patients with the same Gleason score, showed significantly higher intensity of APPL1 staining in the GS ≤ 3 + 4 PCa tissue, compared to the normal tissue (p=0.0366). With increasing grade however, APPL1 seemed to have significantly lower staining intensity, as demonstrated in tissue of GS=4 + 3, when compared to GS ≤ 3 + 4 tissue (p=0.0101). In particular, 3 + 4 tissue had significantly higher staining compared to 4 + 3 tissue (p=0.0248). Overall, APPL1 had a weak to moderate expression in PCa epithelial cells.

Examples of weak to moderate staining, according to the 0–3 scoring system in three distinct examples of tissues stained with MORF4L1 are shown in **Supplementary Figure 4**. In the case of normal compared to cancerous tissue from the same patient, there was a slightly higher MORF4L1 staining in PCa tissue compared to normal one. Statistical analysis in patients with GS=3 + 4 or GS=4 + 3 showed slightly higher MORF4L1 staining intensity, compared to normal tissue, without however reaching significance. GS=4 + 3 patients had slightly higher MORF4L1 staining, compared to GS=3 + 4 ones, which did not reach significance. Therefore, MORF4L1 showed weak to moderate staining in PCa tissue, with a slightly higher intensity in staining based on stage of disease progression. While antibody staining of this TMA for MORFL1 was less successful resulting in low numbers, some correlation was seen for MORF4L1 with Gleason grade albeit with low tumour numbers. We then analysed the TCGA dataset and saw that while miR-1271 mRNA levels tend to decrease with Gleason grade, MORF4L1 mRNA levels showed the opposite trend, supporting an inverse relationship between the miR and target (**Supplementary Figure 5**).

## 3 Discussion

MiRs are considered to be master regulators of gene expression, acting largely through mRNA degradation and translational inhibition (13). Their ability to alter gene expression has made them potential therapeutic targets, or therapeutics, in a number of diseases, including cancer (15, 16). In this study, we explored the role of miR-1271–5p in PCa progression and the effect of altering its levels on AR activity, cell growth, apoptosis and target gene expression.

MiR-96 has been shown to act as an oncomiR in PCa, and miR-1271–5p is a member of the miR-96 family, with the two sharing a similar ‘seed’ sequence, hence indicating similar biological activity (17, 22). MiR-96 has been found to promote bone metastasis in PCa and acts as a biomarker and an indicator of disease progression (23). Conversely, TCGA data shows miR-1271 mRNA is reduced in PCa compared to normal tissue and levels are reduced with Gleason grade (24). Other studies support a potential tumour suppressive effect of miR-1271–5p. In a high throughput analysis, miR-1271–5p was found to be among 18 miRNAs with differential (lower) expression in positive (N1), compared to negative (N0) lymphatic disseminated samples from PCa patients, indicating a potential role of this miR as a prognostic marker for PCa (25). Disheveled-Axin domain containing 1 (DIXDC1) is considered to have an involvement in the development and progression of cancers and was found in a study to be highly expressed in PCa. MiR-1271–5p was found to have an inhibitory effect on DIXDC1 expression, suppressing proliferation, invasion and Wnt signalling in PCa cells (26). The ETS-related gene (ERG), an oncogene often expressed in PCa, was found in another study to be a miR-1271 target gene, with miR-1271 being downregulated in ERG-positive PCa cases (27). Another protein that has been linked to PCa progression, the bromodomain protein TRIM66 (28), has been reported to be a downregulated target of miR-1271–5p, with its expression being reduced when miR-1271 levels were upregulated, in a study of Docetaxel resistant DU145 and PC3 cells (29).

Overall, miR-1271 is involved in regulating multiple signalling pathways, both upstream and downstream. Interestingly, in digestive system tumours, miR-1271 mainly acts on upstream regulators, through epithelial–mesenchymal transition (EMT) pathway, while in tumours of the reproductive and urinary system, including PCa, miR-1271 can both upregulate and donwnregulate gene expression, playing roles in cell proliferation, apoptosis, and invasion (30). Therefore, this investigatory work was an effort to further elucidate the potential effects of miR-1271–5p modulation in PCa.

Our data supports a more complicated role of miR-1271–5p across PCa, depending on tumour stage, from AR dependence to castrate resistance. We have demonstrated that miR-1271 is capable of acting as an oncomiR, promoting cell proliferation in models of hormone-sensitive stages of PCa. MiR-1271–5p inhibition could thus have future therapeutic possibilities. In cells we investigated this by using ASO-1271–5p, which significantly reduced AR-driven reporter gene activity in one out of the two cell lines tested (C42), while the use of Mimic-1271–5p significantly increased reporter gene activity in these cells. In two of six cell lines tested (LNCaP and VCaP), ASO-1271 caused a significant reduction of cell growth, while the mimic had the opposite significant effect in three of six cell lines (LNCaP, C42, VCaP). ASO-1271 significantly increased caspase activity, indicating increased apoptosis, in two of the six cell lines tested (C42, VCaP) and the mimic increased apoptosis in three of six cell lines (22RV1, PNT1A, VCaP). This suggests that miR-1271–5p inhibition with ASOs could potentially be a therapeutic candidate, possibly in combination with current available hormonal treatments, such as enzalutamide, in stages of PCa, prior to hormone resistance.

To begin to elucidate potential pathways by which miR-1271–5p modulation is having its effects, we turned to a number of potential target genes. The transcriptional repressor SPEN, identified by AGO2-PAR-CLIP analysis (21) as targeted by miR-1271–5p, seems to have a protective role in PCa. Loss of SPEN is reported to be oncogenic and SPEN mutations are significantly enriched in metastatic and clonal samples (31). Our preliminary evidence indicates that SPEN is a *bona fide* direct mRNA target of miR-1271–5p, with SPEN mRNA levels having increased significantly, in two different cell lines after addition of ASO-1271–5p. This suggests that a potential oncogenic role of miR-1271–5p could be via downregulation of SPEN, and that ASO-1271–5p could reverse this. The use of ASO-1271–5p in 22RV1 cells similarly led to a significant upregulation of mRNA levels of another candidate target, MORF4L1, while the mimic had the opposite effect. Mortality Factor 4 Like 1 (MORF4L1), also known as MORF-related gene on chromosome 15 (MRG15), is a protein-coding gene and member of the mortality factor (MORF) family of transcription factors, with unique functions, which include interactions with co-repressor complexes (32). MORF4L1 interacts with the BRCA multiprotein complex, and thereby mediates DNA damage response and repair of DNA double-strand breaks, having therefore a potential tumour suppressor role, through interaction with PALB2 and BRCA, members of the DNA damage repair signalling pathway (33). A third potential target, SND1, is a transcriptional coactivator highly expressed in CRPC tissues and has been shown to be overexpressed in PCa (34, 35). In two out of six cell lines, C42 and 22RV1, the addition of ASO-1271–5p led to a significant reduction of SND1 mRNA levels. This would suggest it being a miR-upregulated target, as treating cells with Mimic-1271–5p did result in increased SND1 levels in LNCaP, 22RV1and VCaP cells, with the latter reaching significant levels. At the protein level, SND1 had stronger staining in PCa tissue, compared to normal tissue, with stronger staining seen in higher GS tissue (higher in 4 + 3 tissue, compared with 3 + 3 and 3 + 4 tissue).

Overall, it is demonstrated that *SPEN, SND1* and *MORF4L1* are three target genes of miR-1271–5p with the potential to play significant roles in PCa progression, which could be modified through miR-1271–5p modulation. Further PCa models (*in vivo*, patient serum and explants or organoids) could be investigated in the future to elucidate the complex role of this miR in PCa progression and therapy. PCa, with its heterogeneity and distinct hormonal background, is a complex model, with many molecular interactions. In the future, measurement of the levels of multiple miRs may play a pivotal role in clinical decision making.

## 4 Materials and methods

### 4.1 Mammalian cell culture

Prostate cells used included a representative of normal prostate epithelium (PNT1A) and PCa cell lines, representing distinct phases of disease progression: LNCaP, C42, 22RV1, VCaP and PC3 (details of cell lines are included in **Supplementary Table 1**). LNCaP/MAR4 and C42/MAR4 PCa cells express a stably integrated androgen-responsive ARE-luciferase vector (36). Cells were maintained at 37°C in 5% CO2. LNCaP/MAR4, C42/MAR4 PCa cells were maintained in full growth medium RPMI (Roswell Park Memorial Institute)-1640 Medium (25mM HEPES and NaHCO3) (Sigma Life Sciences, MO, USA), supplemented with 10% tetracycline-free (TF) fetal calf serum (FCS), 100 units/mL penicillin, 100ug/mL streptomycin, 2mM L-glutamine (Sigma Life Sciences, MO, USA), 12ug/mL blasticidin and 500mg/mL G418 (Sigma Life Sciences, MO, USA) to maintain selection for ARE-LUC for both C42/MAR4 and LNCaP/MAR4. For experiments involving androgen treatment, cells were seeded in Minimal Growth Medium (MGM– phenol red-free RPMI supplemented with 5% charcoal-stripped FCS and penicillin, streptomycin and L-glutamine as above). LNCaP, C42, PC3, PNT1A and 22RV1 PCa cells were maintained in full growth medium RPMI (Roswell Park Memorial Institute)-1640 Medium (25mM HEPES and NaHCO3) (Sigma Life Sciences, MO, USA) and VCaP cells in full growth medium DMEM (Dulbecco’s Modified Eagle’s Medium), (Sigma Life Sciences, MO, USA), supplemented with 10% fetal calf serum (FCS), 100units/mL penicillin, 100ug/mL streptomycin and 2mM L-glutamine (Sigma Life Sciences, MO, USA).

### 4.2 Sulphorhodamine B (SRB) assay

LNCaP/MAR4 and C42/MAR4 were seeded in 96-well plates, in 80ul per well antibiotic-free MGM, supplemented with 10nM androgen (mibolerone, MB, PerkinElmer, MA, USA), at dilutions of 4000 and 3000 cells per well, respectively. For 22RV1 and VCaP cell lines, 4000 cells per well were seeded and for PNT1A and PC3 cell lines, 2000 cells per well. Transfection with miR inhibitors (ASOs) was performed 24h post seeding, in a reaction volume of 20ul per well. At days 0, 2 and 6 post-transfection, 100ul of 40% trichloroacetic acid (TCA) was added per well, to fix the cells. The plates were incubated at Room Temperature (RT) for 1h, gently washed with distilled water (x3) and air-dried. Cells were then fixed and stained by addition of 100ul Sulphorodamine-B (0.4% w/v SRB in 1% acetic acid) per well and the plates were left at RT for 1h. Plates were then thoroughly washed 5 times in 1% acetic acid and air-dried. The dried SRB dye was dissolved in 100 ul of 10mM Tris-HCl (pH8.0) and plates were incubated for 1h at RT with shaking. The absorbance was measured at 492nm, using a SunriseTM microplate reader (Tecan, Männedorf, Switzerland). All values were normalised against the day 0 mock-negative control transfection.

### 4.3 Oligonucleotide transfections

MiRCURY LNA (Locked Nucleic Acid) miR inhibitor (ASOs) and mimic (Exiqon) for miR-1271–5p were transfected into cells at 60–80% confluency, in antibiotic-free RPMI, using LipofectamineTM RNAiMAX Reagent (Invitrogen, MA, USA), according to the manufacturer’s instructions, in 6-well plates. Transfection mixes were prepared in Opti-MEM Reduced Serum Medium (Phenol red-, HEPES+, L-Glutamine+) (GibcoR Life Technologies, NY, USA). MiRCURY LNA NC Inhibitor (Exiqon) was used as negative control. SicellDeath (sicDeath), a pool of siRNAs that knock-down various genes essential for cell survival, was used as a positive control (Qiagen) for transfection efficiency (20). Final concentrations of mimics, inhibitors and controls in the proliferation, apoptosis and expression assays were at 0nM and 20nM (AR luciferase assay was performed at a concentration of 2nM and 20nM for miR-inhibitors and 10nM, 50nM for miR mimics). Assays were performed 48h or 72h post-transfection, as indicated in the legend. For combination treatment of ASO-1271–5p or Mimic-1271–5p (Exiqon) with enzalutamide (MDV3100 Selleck chemicals), final concentration of the ASO or mimic was 20nM and of enzalutamide 0.8uM. 0.8% v/v of DMSO was used as a control for enzalutamide, in combination with NC inhibitor or mimic. The concentration of enzalutamide has been previously validated in our lab as effective for maximal inhibition.

### 4.4 AR luciferase reporter assay

LNCaP/MAR4 cells were seeded in stripped, antibiotic-free, MB supplemented RPMI medium. 48h post seeding, ASO-1271–5p and Mimic-1271–5p were transfected (transfection volume: 50ul per well). 48h post transfection, 60ul of reporter lysis buffer was added per well and cells were incubated for minimum 20 minutes at -80C. Plates were thawed on ice and 20ul of cell lysate from each well of the 24-well plate were added in duplicates to wells of a white-walled 96-well Optiplate. 20uL of 2x LucLite substrate (PerkinElmer, MA, USA) was added per well. Plates were incubated at RT in the dark for 15 minutes. Luminescence (counts per second) was assayed, using a VICTOR Light luminescence counter (PerkinElmer, MA, USA) and Wallac 1420 Workstation Software. Luminescence values (RLU; Relative Light Units per second) are proportional to AR luciferase activity.

### 4.5 Luminescent caspase-3/7 Glo assay

C42/MAR4 and LNCaP/MAR4 cells were seeded into white-walled, clear-bottomed 96-well plates, at a density of 8x10^3^ cells and 6x10^3^ cells per well, respectively. PC3 cells were seeded at a density of 6x10^3^ cells per well and PNT1A at 8x10^3^ cells per well. Oligonucleotide transfections were performed using Lipofectamine RNAiMax Reagent (Invitrogen, Paisley, UK). The cells at 60–80% confluency, in antibiotic free RPMI, were transfected with ASO-1271–5p or Mimic-1271–5p, at a final concentration of 20nM. Cells were transfected with MiRCURY LNA NC inhibitor or MiRCURY LNA NC mimic (Exiqon) as negative control and AllStars Cell Death Control siRNA (sicDeath) (Qiagen) as positive control respectively. 72h post-transfection, the cells were allowed to equilibrate to RT for 1h. Caspase 3/7-Glo reagent (Promega, UK) was added (50uL) to each well of the triplicates of the 96-well plates and the content of the plates was gently mixed at 300–500rpm for 30s. Plates were incubated at RT in the dark for 1h and luminescence (counts per second) was measured using a VICTOR Light luminescence counter (PerkinElmer, MA, USA) and Wallac 1420 Workstation Software, to assess apoptosis through caspase activity.

### 4.6 RNA extraction

Total RNA was extracted from cells using Trizol reagent (Invitrogen, MA, USA), according to manufacturer’s instructions. RNA concentration was measured using the NanoDrop (Thermo Fisher Scientific, DE, USA) spectrophotometer and ND-1000 Software.

### 4.7 Real-time qRT-PCR

cDNA was prepared from 500ng total RNA using Precision qScript Reverse Tran-scription kit (PrimerDesign, UK) and oligo d(T) primers. cDNA samples were amplified using 2x Fast SYBR Green Master Mix (SYBR Green I Dye, AmpliTaq Fast DNA Polymerase, Uracil- DNA Glycosylase, ROX dye Passive Reference, dNTPs and optimised buffer components) (Applied Biosystems, MA, USA) and 250nM forward and reverse primers (**Supplementary Table 2**). The 7900HT Fast RT-PCR System (Applied Biosystems, MA, USA) was used. PCR parameters were set at 95°C for 20s, followed by 40 cycles of 95°C for 1s and 60°C for 20s. Primer efficiency was tested with standard curves and an additional cycle of 95°C for 15s, 60°C for 15s and 95°C for 15s was performed to obtain dissociation curves for each primer pair. Data was recorded using the Sequence Detection System. All data were analysed using the ΔΔCt method. All mRNA levels were normalised to ribosomal protein L19 mRNA levels.

### 4.8 Statistical analysis

Normally distributed continuous variables were assessed by an unpaired (two sample) Student’s t-test (two tailed), and P ≤ 0.05 was interpreted to denote statistical significance. Where no asterisks are given, difference is not significant. For immunohistochemistry assays where many variables are included, such as different patients with various Gleason scoring patterns, as well as staining scores that vary between antibodies, one-way ANOVA test has been used, to give substantial significance between various comparisons among the different groups.

### 4.9 Immunohistochemistry

Tissue microarrays (TMAs) with samples from a total of 61 PCa patients on four arrays were provided by the Imperial Cancer Biomarker Resource Centre (ICBRC), after approval from Imperial CRUK Steering Committee, (REC: 12/WA/0196). Prostate samples were collected after radical transurethral resection of the prostate (TURP). A total of four sections were provided per patient, two sections with benign tissue and two sections with cancer. Details of the TMA demographics and clinical characteristics are given in **Supplementary Table 3**. Adjacent sections were subjected to staining using antibodies for SND1 (1:200 dilution). Details of the SND1, APPL1 and MORF4L1 antibodies used for immunohistochemistry can be found in **Supplementary Table 4**. Immunohistochemistry was performed following standard protocol (details in S.1 Materials and Methods: Immunohistochemistry protocol). Pictures were taken using a Leica DM750 microscope at different magnifications (10x to 20x). For percentage of stained cells, number of cells were counted per field, to determine the number of positive and negative cells. Three different images were taken per tissue, per patient, scored by two independent assessors, following the quick-score staining system, to assess intensity of staining. Statistical analysis was conducted using one-way ANOVA.

## 5 Conclusion

This paper demonstrates that miR-1271–5p has properties of an oncomiR in PCa and the potential of targeting this miR in PCa treatment. We have shown that miR-1271–5p inhibition led to a decrease in AR activity and miR-1271–5p promoted cell growth in AR-positive PCa cell lines, LNCaP, C42 and VCaP. MiR-1271–5p inhibition reduced growth in LNCaP and VCaP cell lines. ASO-1271–5p, in combination with enzalutamide, led to a further reduction of LNCaP cell growth, compared to the effect of enzalutamide alone. These results demonstrate a potential role for miR-1271–5p in the modulation of AR activity and PCa cell growth, variable in relation to miR and AR expression levels between cell lines. We report that the effects of miR-1271–5p inhibition on cell growth correlates with the AR status of PCa cell lines. This suggests that miR-1271–5p may have a key role in the development of the hormone-sensitive disease stage via specific target genes and that ASO-1271–5p could potentially be a therapeutic candidate, possibly in combination with current available hormonal treatments in the setting of hormone sensitive PCa.

## Data Availability

The raw data supporting the conclusions of this article will be made available by the authors, without undue reservation.
