# Supplementary material for: Androgen receptor modulatory miR-1271-5p can promote hormone sensitive prostate cancer cell growth
Source: Front Oncol. 2024 Aug 29;14:1440612. doi: 10.3389/fonc.2024.1440612 (PMC11390458; doi:10.3389/fonc.2024.1440612)
Supplement: Supplementary file 1 [file DataSheet1.pdf]

Supplementary Material

Supplementary Materials & Methods

S.1 Materials and Methods: Detailed Immunohistochemistry Protocol

For deparaffinisation purposes, the slides were washed in Xylene/Histoclear twice, 10min each wash. They were further hydrated with 100% Ethanol, 2 washes for 5min each. Additional washes were conducted using 95% ethanol for 5min and 70% ethanol for 5min. Slides were washed in distilled water, making sure that there was no wax residue remaining, otherwise the previous process was repeated. Further to that, HIER buffer was made (Heat Induced Antigen Retrieval), consisting of 0.01M Sodium Citrate Buffer, pH 6.0 buffer, 2.94g of Tri-Sodium citrate, 1000ml of distilled water, adjusted to a total pH of 6.0, using 1M HCL. Slides were immersed into a container with HIER buffer and the lid was placed loosely on top, at the centre of the microwave. A container was also placed in the microwave, partially filled with water next to the container hosting the slides. Slides were microwaved at high (100%) power for 4mins (or until boiling) and the power was then reduced to a medium level (50%) for an additional period of 10mins. The containers were removed afterwards from the microwave and were allowed to cool down for 15-20min. The slides were then washed twice for 2-5min in PBS. Endogenous peroxidase was blocked, using 3% hydrogen peroxide in PBS for 5 min. Slides were washed in PBS and then blocked with milk for 20min in a humid chamber. The primary antibody was placed on top of the slides, after being diluted, in order to reach the required concentration in the final blocking solution. The blocking solution was carefully placed drop-wise on top of the slides in a humid chamber and was incubated overnight at 4°C. The following day, the slides were washed 3 times, 5min per wash, in PBS. The secondary antibody, provided by the kit (Immunostain kit, Invitrogen, Life Technologies, as per manufacturer), was then placed on top of the slides. The tissue was incubated at RT, for 1h. The slides were washed again 3 times, 5min each, in PBS. Streptavidin was added drop-wise on top of the tissue slides with an incubation time of 10-15min in RT, as per manufacturer's instructions. Slides were further washed 3 times in PBS, 5min each. Antibodies were possible to be visualised using DAB+, as recommended by the manufacturer, after being stained for up to 10min, with constant monitoring, since excessive time of exposure to DAB+ could lead to overstaining. Slides were washed again three times, 5min each, in PBS. Further counterstaining was conducted with the use of Haematoxylin, for 10-30sec. Slides were rinsed with tap water

and further dehydrated through graded dilutions of Ethanol: 50%, 70%, 95% twice and 100% twice as well, 5min per wash. Finally, the slides were immersed in Xylene/Histoclear to avoid any remaining water. Slides were covered and were left to set (refer to standard protocol)

S.2 Materials & Methods: MiRCURY qRT-PCR (for endogenous miR-1271-5p levels)

CDNA was prepared from 10ng total RNA using Universal cDNA synthesis kit II (Exiquon). RT reaction mix included the following reagents per sample: 2ul 5x Reaction Buffer, 1ul Enzyme mix, 0.5ul UniSp6 spike-in, 4.5ul water. The thermal cycle for the RT was set at 42°C for 60min (Polyadenylation-cDNA synthesis step) and 95°C for 5min (for Heat inactivation of the RTase). cDNAs were further diluted 80x in water + 1:20 ROX. The final concentration of ROX in each well was at 500nM (diluted 1:50). cDNAs were amplified using ExiLENT SYBR Green master mix (Exiquon) with the 7900HT RT-PCR System (Applied Biosystems, MA, USA). PCR parameters were set at 95°C for 10min, followed by 40 cycles at 95°C for 1min and 60°C for 1min. A melting curve analysis was also performed at the end of the 40th cycle. Data were recorded using the Sequence Detection System. All data were analysed using the ΔΔCt method. Endogenous levels of miRs on the cell lines were normalised to the geomean of control primers: U6 and SNORD48.

Supplementary Table 1: Prostate cancer cell lines/general characteristics [1, 2]

Cell lines	PNT1A	LNCaP (parental)	C42	22RV1	PC3	VCaP
**Origin**	Derived from a deceased 35-year-old organ donor-Established by Cussenot et.al. (1991)[3] with SV40 immortalisation of normal prostate epithelium	Lymph node metastatic lesion from a 50-year old Caucasian male	Derived from LNCaP-injected with human MS fibroblast cells into nude mice that were then castrated	xenograft line CWR22R (previously taken from a primary prostatic carcinoma, Gleason score 9, from a patient with osseous metastases	Lumbar vertebral metastasis	Derived from lumbar vertebral metastatic lesion. Tissue xenografted into SCID mice.

**Androgen dependence**	AR independent	AR dependent	AR independent	Both AR dependent and independent features	AR independent	AR dependent
**Receptor status**	AR null<br>No PSA	AR mutant (T877A)<br>PSA positive	AR mutant (T877A)<br>PSA positive	AR mutant (H874Y)<br>PSA positive	AR null<br>No PSA	AR mutant<br>AR amplification<br>PSA positive
**Doubling time**	40 hrs	60 hrs	48 hrs	35-40 hrs	30-40 hrs	53 hrs

Supplementary Table 2: Primers for RT-qPCR

**Amplification Product**	**Primer direction**	**Primer Sequences**
TMBIM6	For	5'-CATATAACCCCGTCAACGCAG-3'
	Rev	5'-GCAGCCGCCACAAACATAC-3'
ELK4	For	5'-TGTCACGACACCTTCCAAAAA-3'
	Rev	5'-GGGGAAACCAATGTCTCCAAAG-3'
APPL1	For	5'-AGAGACCATGCAACAGACAATAG-3'
	Rev	5'GTATCCAGCCTTTCGGGTTAAA-3'
MORF4L1	For	5'-AGCAATGTTGGCTTATACACCTC-3'
	Rev	5'-AGCTTTCCGATGGTACTCAGG-3'
SND1	For	5'-GAGTATGGCATGATCTACCTTGG-3'
	Rev	5'-GCCGGTTCTGCTCAGGATT-3'
SPEN	For	5'-AACTCGGTCAACAAAATGGGT-3'
	Rev	5'-AAACCTCTCTACTACGAGATGCT-3'
L19	For	5' GCAGCCGGCGCAAA 3'
	Rev	5' GCGGAAGGGTACAGCCAAT 3'

Supplementary Table 3: TMA demographics and clinical characteristics

TMA characteristics	Number of patients
Cohort	61
Overall sections per patient	4
Tumour sections per patient	2
Normal sections per patient	2
Low-moderate grade prostate cancer (GS<=3+4)	43
(of which GS=3+4)	38
Moderate grade prostate cancer (GS=4+3)	18
Acute/chronic prostate inflammation	23
(of which) Stromal inflammation	15
PIN	22
(of which) Lymphovascular invasion	4

Supplementary Table 4: Antibodies for immunohistochemistry

	Protein	Protein Size (kDa)	Species	Dilution	Manufacturer	Product Number
**Antibodies for MiR-1271-5p target genes**	SND1	102kDa	Rabbit Polyclonal	1/1000	Abcam (UK)	Ab65078
	APPL1	100kDa	Mouse (Monoclonal)	1/100	Santa-Cruz (USA)	Sc-271909
	MORF4L1	45kDa	Mouse Monoclonal	1/100	Santa Cruz (USA)	Sc-514877

Supplementary Table 5: MiR primers for MiRCURY RT-PCR

Primer mix	Product Number (Exiquon)
miR-1271-5p	204351
U6	203907

SNORD48	203903
Negative control inhibitor	4100001-4104908-001
Negative control mimic	470000-479000-001

Supplementary Table 6: Primers for RT-qPCR

**Amplification Product**	**Primer direction**	**Primer Sequences**
AR	For	5’ CGCGACTACTACAACTTTCCACTGG 3’
	Rev	5’ ACCACCACACGGTCCATACAACTGG 3’
TMPRSS2	For	5’AATCGGTGTGTTCGCCTCTAC 3’
	Rev	5’ GCGGCTGTCACGATCC 3’
PSA	For	5’ TTGTCTTCCTCACCCTGTCC 3’
	Rev	5’ AGCTGTGGCTGACCTGAAAT 3’
DKK1	For	5’-CCTTGGATGGGTATTCCAGA-3’
	Rev	5’-CAGTCTGATGACCGGAGACA-3’

Supplementary Results

Effect of miR-1271-5p on mRNA levels of AR and AR specific target genes

Given that miR-1271-5p was identified as altering AR activity in the initial screen, RT-qPCR assays were used to demonstrate the potential effects of miR-1271-5p manipulation on alteration of mRNA levels of *AR* and its target genes. In LNCaP/MAR4 cells, the use of miR-1271-5p inhibitor did not show an effect on mRNA levels of *AR* compared to negative control, while the use of the mimic slightly decreased the *AR* mRNA levels, when compared to negative control (Supplementary Figure 1). Conversely, mRNA levels of the *AR* target *PSA* were decreased with the use of the inhibitor, while the mimic had the opposite, but non-significant effect, increasing *PSA* mRNA levels. The mRNA levels of *TMPRSS2* were increased, when transfected with miR-1271-5p inhibitor, compared to negative control, without reaching significance, while the addition of miR-1271-5p mimic did not have an effect compared to negative control. MiR-1271-5p mimic slightly decreased *AR*, while the most marked (non-

significant) effects were the decrease of *DKK1* and increase of *TMPRSS2* levels with the inhibitor (Supplementary Figure 1 A).

In the C42/MAR4 cell line, the effects were more marked, with miR-1271-5p inhibitor significantly reducing mRNA levels of the *AR* (p=0.00094) and the *AR* upregulated target genes *PSA* (p=0.0249) and *TMPRSS2* (p=0.0015). The opposite effect was demonstrated for mRNA levels of the *AR* downregulated target *DKK1*, without however reaching significance. Intriguingly, addition of miR-1271-5p mimic to C42 cells also reduced *AR* mRNA levels (p=0.0003), as well as the *AR* target genes *PSA* (p=0.00091) and *TMPRSS2* (p<0.0001), including the downregulating *DKK1* (p=0.0050) (Supplementary Figure 1 B).

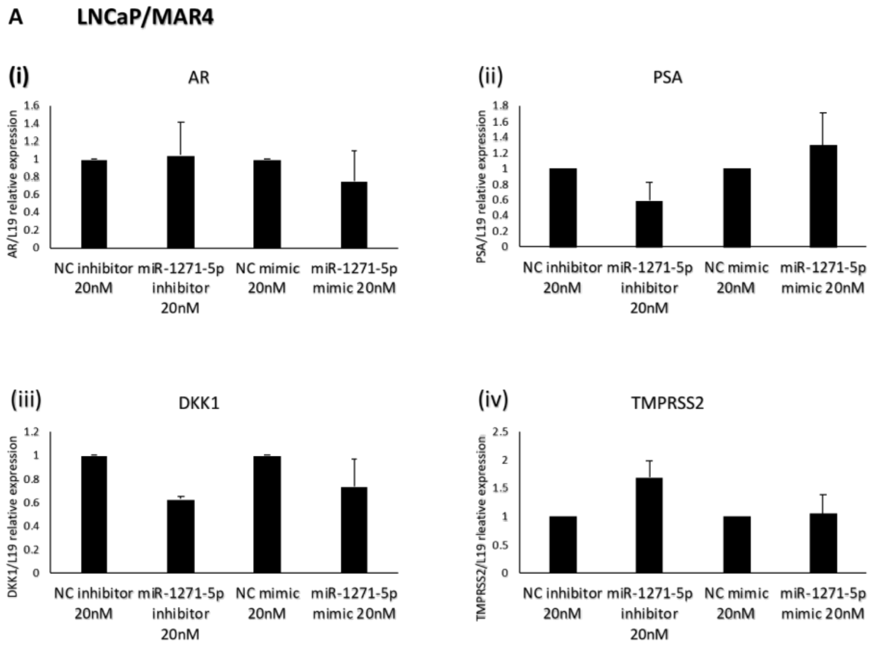

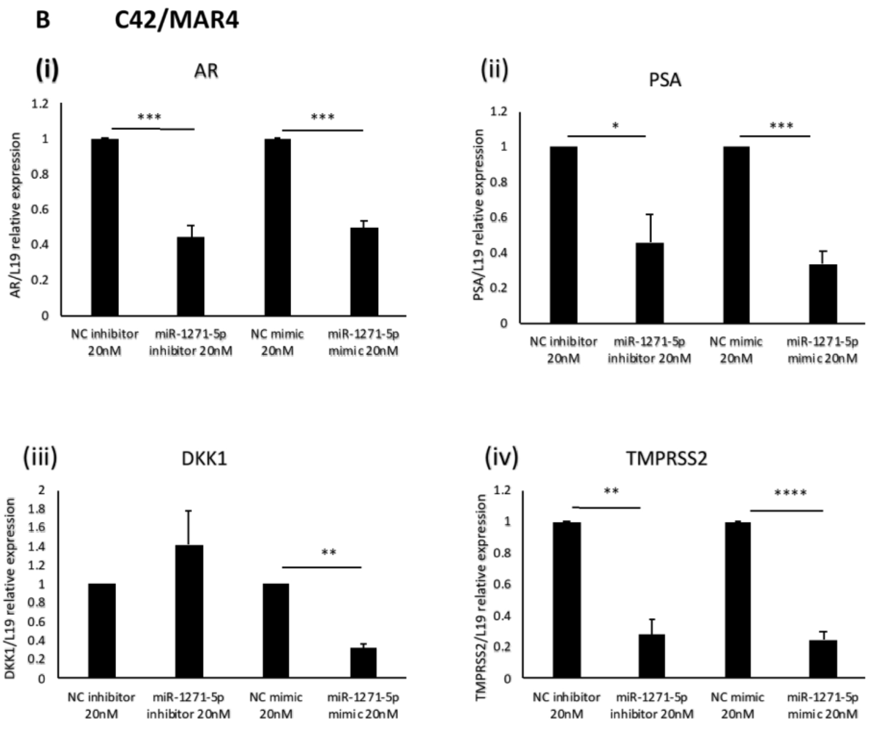

Supplementary Figure 1: Effect of miR-1271-5p inhibitor and mimic on mRNA levels of AR and AR target genes (A): LNCaP/MAR4, (B): C42/MAR4 cells

*RT-qPCR analysis of (i) AR, (ii) PSA, (iii) DKK1, (iv) TMPRSS2 in (A) LNCaP/MAR4 and (B) C42/MAR4 cells transfected with miR-1271-5p inhibitor or mimic at 20nM concentration. Cells were transfected with miR-inhibitor or mimic 24h post seeding and they were further incubated for 48h after the transfection. A negative control was used for each experiment. L19 was used for normalisation. Data represent mean relative expression of three independent experiments performed in triplicate ± SEM. Statistical analysis was performed by an unpaired (two sample) Student's t-test (two tailed). * $P \leq 0.05$, ** $P \leq 0.01$, *** $P \leq 0.001$, **** $P \leq 0.0001$.*

Endogenous levels of miR-1271-5p in prostate cancer cell lines

Endogenous miR-1271-5p expression levels were investigated in order to ascertain whether miR-1271-5p is detectable in prostate cancer cell lines and to determine the most suitable cell lines for subsequent overexpression studies. MiRCURY RT-qPCR was performed, an optimised method for miR amplification.

Generated Ct values were normalised to the average of two controls, the non-coding small nuclear RNA U6 and the small nucleolar RNA SNORD48, which is affiliated to the non-coding RNA class (Supplementary Figure 2). Normalisation of Ct values for miR-1271-5p levels per cell line were conducted with the use of the geomean of U6 / SNORD48, then predicted relative

to the value for the PNT1A cell line set at 1. Relative to U6/SNORD48, values for DU145 (p=0.0038) and VCaP cells (p=0.0280) were significantly higher than the values of PNT1A, followed by 22RV1 cells (Supplementary Figure 2).

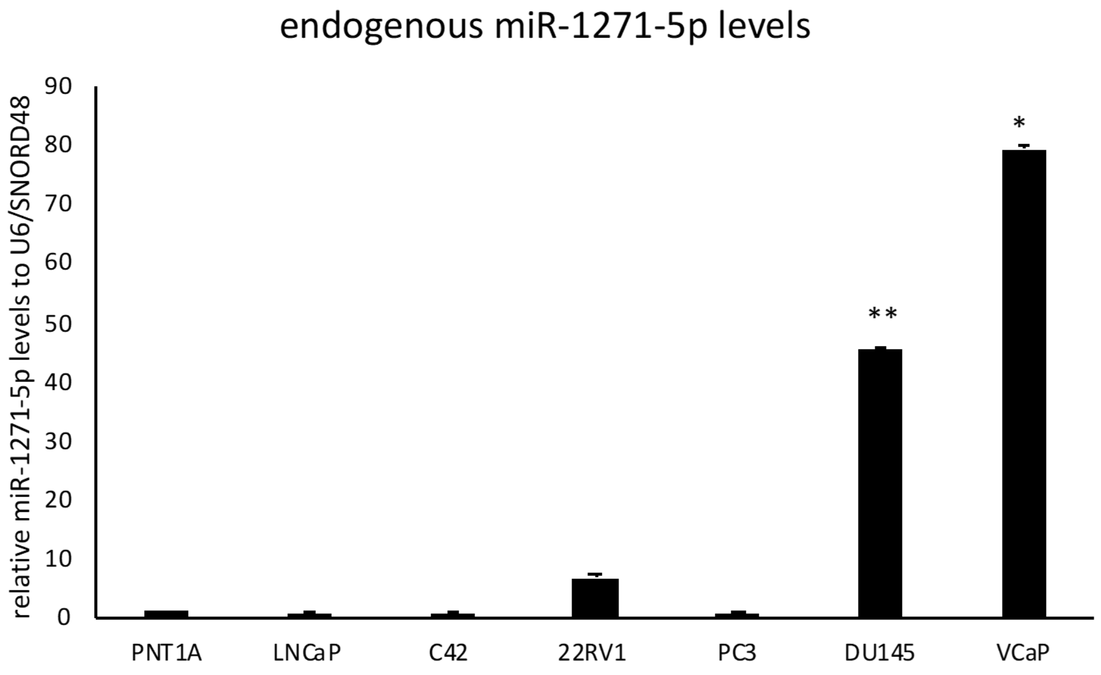

Supplementary Figure 2: Levels of miR-1271-5p on a panel of prostate cancer cell lines

*MiRCURY qRT-PCR analysis of miR-1271-5p expression levels on a panel of prostate cancer cell lines. Data represent the mean relative to PNT1A expression of three independent experiments performed in triplicate ± SEM. Normalisation was conducted with the use of geomean U6 and SNORD48. Statistical analysis was performed by an unpaired (two sample) Student's t-test (two tailed, relative to the normal prostate epithelial cell PNT1A). * P ≤ 0.05, ** P ≤ 0.01.*

Expression of miR-1271-5p Target Gene APPL1 in PCa Tumours

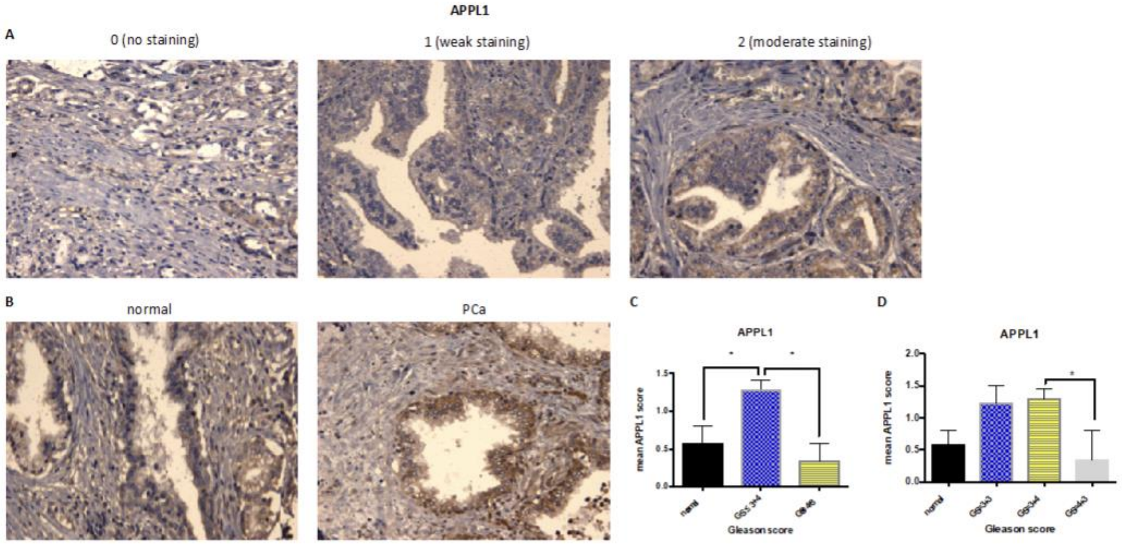

Supplementary Figure 3: APPL1 staining is weak to moderate in PCa tissue.

*(A): Examples of APPL1 immunohistochemistry in epithelial cells using the quickscore method as : 0 (negative), 1 (weak), 2 (moderate) staining. (B): Benign and cancer sections from the same patient were stained for APPL1. (C),(D): Statistical analysis of the mean APPL1 staining intensity, based on the 0-3 scoring system, relative to Gleason Score. Data represent mean values of scores from patients grouped based on Gleason score from three TMAs used. Normal: n=51, GS≤3+4: n=36, with GS=3+3: n=5 and GS=3+4: n=31, GS=4+3: n=15. Three different images were taken per tissue, per patient, scored by two independent assessors. Statistical analysis was conducted using one-way ANOVA. * $P \leq 0.05$, ** $P \leq 0.01$, *** $P \leq 0.001$, **** $P \leq 0.0001$.*

Expression of miR-1271-5p Target Gene MORF4L1 in PCa Tumours

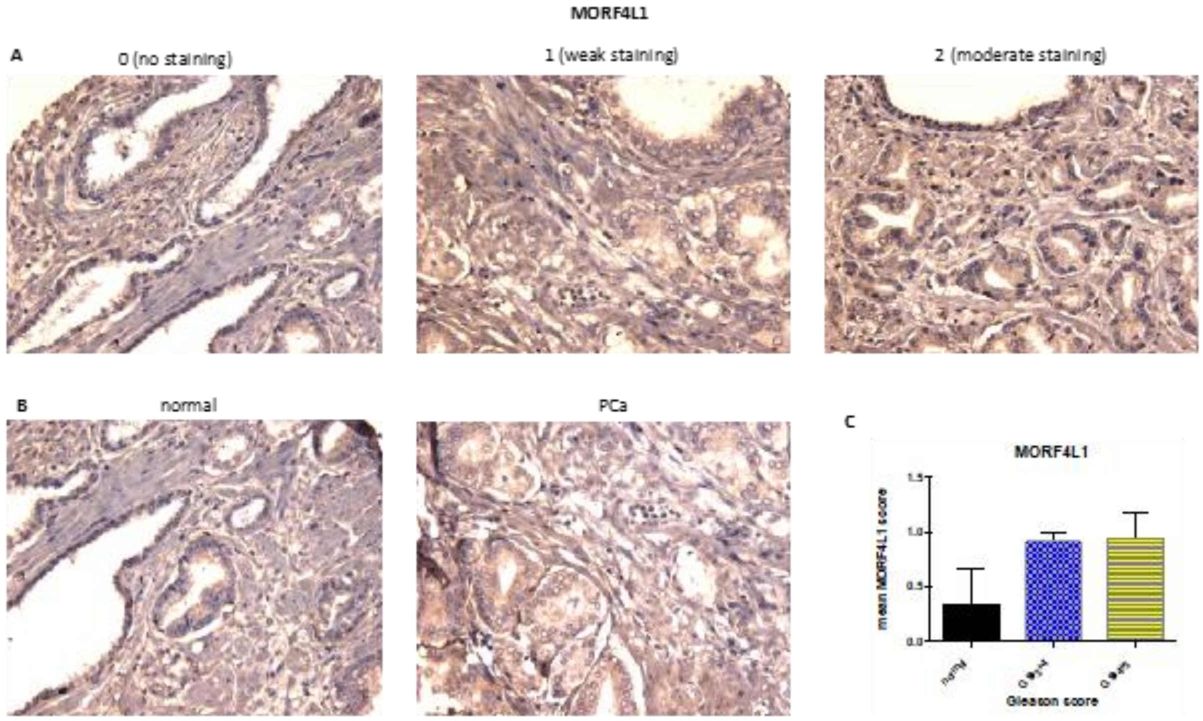

Supplementary Figure 4: MORF4L1 staining is weak to moderate in prostate cancer tissue

(A): Examples of MORF4L1 immunohistochemistry in epithelial cells using the quickscore method as : 0 (negative), 1 (weak), 2 (moderate) staining. (B): Benign and cancer sections from the same patient were stained for MORF4L1. (C), (D): Statistical analysis of the mean MORF4L1 staining intensity, based on the 0-3 scoring system, relative to Gleason score. Data represent mean values of scores from patients grouped based on Gleason score. Normal: n=10, GS=3+4: n=7, GS=4+3: n=3. Three different images were taken per tissue, per patient, scored by two independent assessors. Statistical analysis was conducted using one-way ANOVA.

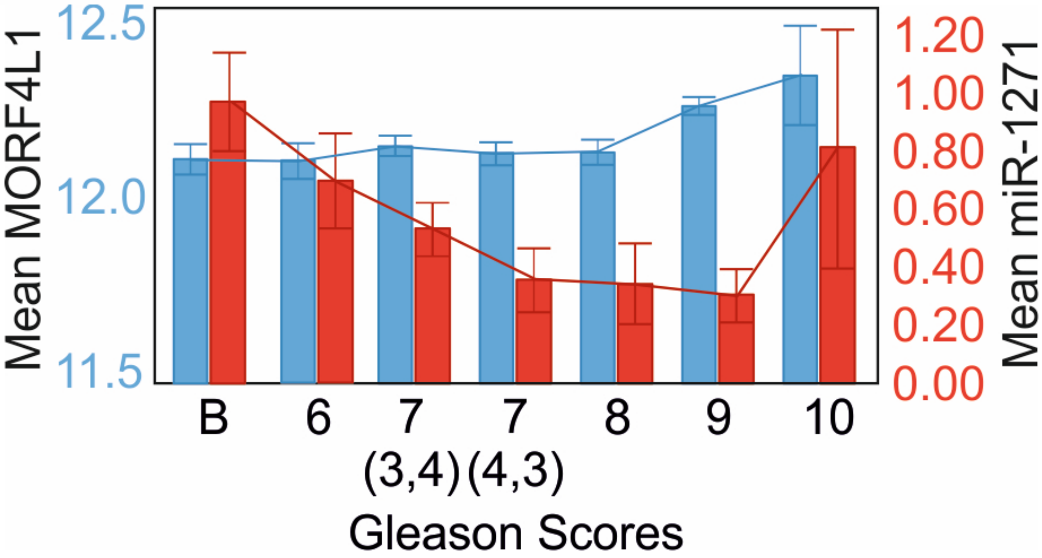

Supplementary Figure 5: MORF4L1 mRNA shows opposite trend to miR-1271 expression in TCGA dataset

Log2 expression data of MORF4L1 mRNA (blue columns, left Y-axis) and miR-1271 (red columns, right Y-axis), represented as means with SEM error bars. X-axis represents Benign samples (n=50) and samples from different Gleason Scores (G6 n=44, G7(3,4) n=146, G7(4,3) n=101, G8 n=64, G9 n=138, and G10 n=4. Note the very low numbers for Gleason grade = 10 may skew the final datapoint.

References

1. Tepper, C.G., et al., *Characterization of a Novel Androgen Receptor Mutation in a Relapsed CWR22 Prostate Cancer Xenograft and Cell Line*. Cancer Research, 2002. **62**(22): p. 6606-6614.
2. Sobel, R. and M. Sadar, *Cell lines used in prostate cancer research: a compendium of old and new lines—part 1*. The Journal of urology, 2005. **173**(2): p. 342-359
3. Cussenot O, Berthon P, Berger R, Mowszowicz I, Faille A, Hojman F, Teillac P, Le Duc A, Calvo F. *Immortalization of human adult normal prostatic epithelial cells by liposomes containing large T-SV40 gene*. J Urol. 1991 Sep;146(3):881-6. doi: 10.1016/s0022-5347(17)37953-3. PMID: 1714974.
